# IL-17A Cytokine-Regulated Glut1 Expression in Placenta Cells

**DOI:** 10.3390/cimb46070438

**Published:** 2024-07-12

**Authors:** Jeong Yeon Lee, Hyunju Kim

**Affiliations:** Department of Health Sciences, The Graduate School of Dong-A University, Busan 49315, Republic of Korea; pdc06057@naver.com

**Keywords:** IL-17A, Glut1, placenta, maternal immune activation, gene expression

## Abstract

Trophoblasts, the principal cellular component of the placenta, play an important role in nutrient and gas exchange. Previous studies have indicated that maternal immune activation (MIA) leads to an elevation in IL-17A cytokine levels in maternal serum, subsequently influencing fetal brain development during pregnancy. In this study, we aimed to elucidate the impact of the IL-17A cytokine on placental function. First, we treated JAR and JEG-3, which is a placenta cell line, with IL-17A in a concentration-dependent or time-dependent manner and observed cell morphology and viability. It was confirmed that treatment with IL-17A or a double-stranded RNA mimic (PolyI:C) had no effect on the morphology or cell viability. IL-17A treatment increased the expression of IL-17R at the mRNA and protein levels, and Poly(I:C) increased the levels of IFNγ and TNFα. Additionally, PPARγ, known as a metabolism regulator, was increased by IL-17A treatment. Also, we observed that the expression of Glut1 and Glut3 was increased by IL-17A treatment. To confirm this, we examined the expression of transporters in the placental tissue of the MIA rodent model, and we observed that mRNA expression of glut1 and glut3 was significantly increased. However, the expression of Gltu1 and Glut3 was observed to be significantly inhibited in the brains of MIA-induced offspring. This study suggests that IL-17A increases signaling through IL-17R in the placenta and fetal brain tissue; however, there is a mechanism for regulating the expression of glucose transporters by increased IL-17A in the placenta.

## 1. Introduction

The placenta, which is responsible for the exchange of nutrients, gases, and waste products between the physically separate maternal and fetal circulations, is an organ that plays an important role in supporting the growth and development of the fetus during pregnancy [1,2]. Additionally, the interactions of various cytokines, immune cells, and factors within the placental microenvironment have been reported to influence pregnancy outcomes [3,4]. The pro-inflammatory cytokine interleukin-17A (IL-17A) is produced primarily by CD4^+^ T cells (Th17 cells) and subsets of other immune cells, such as γδT cells, natural killer cells (NK cells), and certain innate lymphoid cells (ILCs). After binding to the IL-17 receptor (IL-17R), IL-17A is functionally involved in the pathogenesis of several autoimmune diseases, such as psoriasis, rheumatoid arthritis (RA), lupus, inflammatory bowel disease (IBD), and multiple sclerosis (MS) [5]. Some studies have reported that IL-17R is expressed in placental tissue, and IL-17A-IL-17R signaling plays an important role in regulating immune responses and inflammation at the maternal–fetal interface during pregnancy [6,7,8]. Furthermore, elevated IL-17A levels in the placenta, amniotic fluid, and maternal serum have been reported to increase the risk of preterm birth [9]. However, the regulatory mechanisms of IL-17A in the placenta and the specific role of IL-17A during pregnancy are complex and are not yet fully understood.

The placenta contains a diverse group of proteins known as placental transporters, which play an important role in facilitating the exchange of various substances between maternal and fetal circulation [2]. The expression and activity of placental transporters are influenced by a variety of factors, including the stage of pregnancy, maternal health, and existing medical conditions, and the disruption of transporter function is known to result in pregnancy complications or fetal development problems [10]. Glucose, which serves as the main source of energy for the fetus, is obtained through maternal circulation because the uterus does not obtain enough glucose for growth through gluconeogenesis [11,12,13]. Therefore, the glucose transporters present in the placenta are mainly regulated by the maternal circulation, which is mainly regulated by the maternal-to-fetal concentration gradient, placental cell metabolism, and GLUT1 expression and activity in the placenta.

Neurodevelopmental disorders such as autism spectrum disorder (ASD) are associated with increased levels of IL-6, IL-17, and tumor necrosis factor (TNF), and glut1 deficiency is known to contribute to cognitive impairment, motor problems, epilepsy, and Alzheimer’s disease [14,15]. Therefore, in this study, we investigated the expression of various transporters in placenta cells by IL-17A treatment and confirmed it through an ASD rodent model.

## 2. Materials and Methods

### 2.1. Cell Culture and IL-17A Cytokine Treatment

Human trophoblast cell lines, JAR and JEG-3 (#30144, #30036, respectively, purchased from Korean cell line bank (KCLB)) were cultured in Dulbecco’s Modified Eagle’s Medium (DMEM, Welgene, Kyoungsan, Republic of Korea, #LM001) containing 10% heat-inactivated fetal bovine serum (FBS, Welgene, #S001) and 1% penicillin/streptomycin (P/S, Welgene, Kyoungsan, Republic of Korea, #LS202). These cells were maintained at 37 °C in humidified 5% CO_2_ incubators. The medium was replaced every 2 days.

### 2.2. MTT Assay and Cell Proliferation Assay

For measuring cell viability, we performed an MTT (3-(4,5-Dimethylthiazol-2-yl)-2,5-Diphenyltetrazolium bromide) assay (Invitrogen, Waltham, MA, USA, #M6494). Cells were seeded at a density of 1 × 10^4^ cells/well into a 96-well plate and incubated with 100 µg/mL of polyinosinic: polycytidylic acid I:C (Poly(I:C)), Sigma-Aldrich St. Louis, MO, USA, #P9582) or 100 ng/mL of recombinant human IL-17A (PeproTech, Waltham, MA, USA, #200-17). After 24 h, supernatants were discarded, and cells were incubated with 100 µL of 0.3 mg/mL MTT solution in the medium for 30 min in the incubator at 37 °C. Then, media were replaced with 200 µL of dimethyl sulfoxide (DMSO), and optic density (O.D.) values were measured at 540 nm using a microplate reader (Bio-Rad, Hercules, CA, USA).

To measure cell proliferation, cells were seeded at a density of 1 × 10^5^ cells/well in a 6-well plate and incubated with Poly(I:C) at 0, 10, 50, and 100 µg/mL or IL-17A at 1, 10, 50, and 100 ng/mL for 24 and 48 h. At different time points, cells were harvested and stained with trypan blue and then counted using a hemocytometer under a microscope.

### 2.3. Reverse Transcriptase PCR (RT-PCR)

Cells were seeded into a 6-well plate at a density of 3 × 10^5^ cells/well and incubated with Poly(I:C) or IL-17A at 100 µg/mL or 100 ng/mL for 24 h and were harvested. Total RNA was isolated from the placenta and fetal brain of mice at the age of embryonic E18.5 or from JAR and JEG-3 cells using Trizol (Takara, Kusatsu, Shiga, Japan, #9109) according to the manufacturer’s protocol. An RNA of 2 µg was used to synthesize cDNA with an oligo dT primer (Genet Bio, Daejeon, Republic of Korea, #SR-5000) and followed by being reversely transcribed to cDNA with a transcript cDNA synthesis kit according to the manufacturer’s instructions. The sequences of the primers used for RT-PCR experiments were as follows [Table 1]:

### 2.4. Western Blotting

Cells were seeded into 100 mm dishes at a density of 1 × 10^6^ cells/dish and incubated with poly(I:C) at 100 µg/mL or 100 ng/mL IL-17A for 24 h. After being treated with reagents, cells were lysed with RIPA lysis buffer (Thermo Fisher Scientific, Waltham, MA, USA, #89900) containing a protease inhibitor cocktail (Thermo Fisher Scientific, #1862209) and a phosphatase inhibitor cocktail (Thermo Fisher Scientific, #1862495). Following centrifugation at 13,000× *g* for 20 min at 4 °C, the supernatant was collected and quantified using a BCA assay kit (Thermo Fisher Scientific, #23225). Total protein lysates were separated by sodium dodeoxyl-sulfate polyacrylamide gel electrophoresis (SDS-PAGE) and transferred to a polyvinylidene fluoride (PVDF) membrane (Millipore, Burlington, MA, USA, #IPVH00010). The membrane was blocked with 5% non-fat milk in tris-buffered saline (TBS) containing 0.1% tween 20 (TBST) for 1 h at RT and then incubated with primary antibodies at 4 °C overnight (O/N). Primary antibodies used in this study included anti-IL-17RA (Cell Signaling, Danvers, MA, USA, cs-12661), anti-PPARγ (Cell Signaling, cs-2443), anti-ZO-1 (Santa Cruz, Dallas, TX, USA, sc-33725), and anti-GAPDH (Santa Cruz, sc-32233). Following incubation, the membrane was washed 3 times for 10 min in TBST and incubated with an HRP-conjugated secondary antibody for 1 h at RT following three 10 min washes. Secondary antibodies used were anti-mouse (Cell Signaling, cs-7076) and anti-rabbit (Cell Signaling, cs-7074). Detection was performed using X-ray films and enhanced chemiluminescence (ECL) reagents (Millipore, #212185).

### 2.5. Maternal Immune Activation (MIA)

Female and male (C57BL/6) mice (8 to 12 weeks old) were obtained from Hana-Biotech and housed at 20 °C on a 12 h light/dark cycle with *ad libitum* access to food and water. All animal experiments were approved by the Institutional Animal Care and Use Committee of Dong-A University (the approved number is DIACUC-23-41). Eight-to-twelve-week-old C57BL/6 females and male mice were mated at a ratio of 2:1. The next day, a vaginal plug occurred on embryonic day 0.5 (E0.5). At E12.5, pregnant mice were intraperitoneally (i.p.) injected with 20 mg/kg (dissolved in sterile phosphate buffer saline (PBS)) of the viral mimetic Poly(I:C) (potassium salt, Sigma-Aldrich, #9582) or vehicle (PBS). Pregnant females were randomly assigned to either the vehicle or the Poly(I:C) group. Mice were sacrificed at E18.5, and placenta and fetal brains were obtained. RNA was isolated using TRizole solution (Thermo Fisher Scientific, Waltham, MA, USA #15596026), and RT-PCR was performed using each primer [Table 2]. Genomic DNA isolated from the fetus tail was used to determine the gender of each pup by PCR using primers annealing to the Y chromosome SRY gene (forward-CAAGTTGGCCCAGCAGAATC, reverse-TGTGACACTTTAGCCCTCCG).

### 2.6. Statistical Analysis

All data were analyzed using GraphPad Prism 5. Differences between groups were evaluated using a *t*-test, a one-way analysis of variance (ANOVA), and a two-way ANOVA test. Significance levels were indicated as follows: * *p* < 0.05, ** *p* < 0.01, *** *p* < 0.001.

## 3. Results

**Figure 1:**
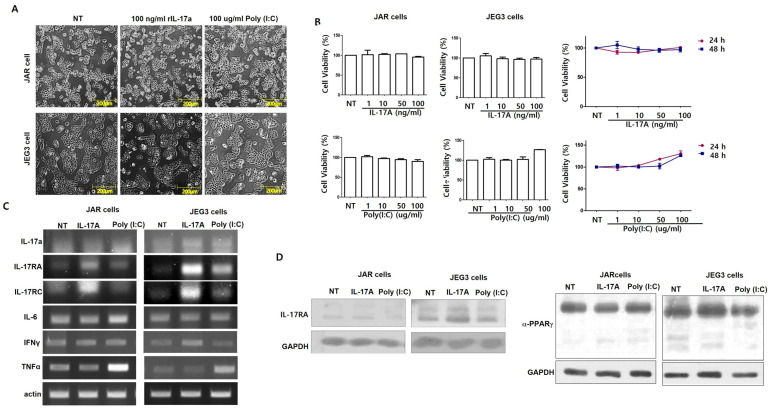
IL-17A or Poly(I:C) treatment of the placenta cell lines. Cell morphologies were investigated by IL-17A or Poly(I:C) treatment for 24 h (**A**). Cell viabilities were measured in a dose-dependent manner for 24 h and in a time-dependent manner by MTT assay (**B**). Activation of IL-17RA, IL-17RC, IL-6, IFNγ, and TNFα mRNA expression in placental cell lines (**C**). IL-17RA, PPARγ protein expression in cells treated with 100 ng/mL IL-17A or 100 µg/mL Poly(I:C) (**D**). GAPDH was used for loading control. Calculated by *t*-test (**B**,**C**) or one-way ANOVA with Tukey’s test.

To investigate the effect of IL-17A cytokine on placental cells (JAR and JEG-3 cells), we administered recombinant human IL-17A cytokine or Poly(I:C), a synthetic analog of double-stranded RNA (dsRNA), to the cells. Cell morphology and viability were observed at various concentrations for 24 h. IL-17A cytokine was treated from a low concentration of 1 ng/mL to a high concentration of 100 ng/mL, and Poly(I:C) was used at a concentration of 1~100 μg/mL. Treatment with IL-17A or Poly(I:C) did not induce morphological changes in JAR and JEG-3 cells [Figure 1A]. Also, the MTT assay results confirmed that treatment with IL-17A or Poly(I:C) did not induce cell death [Figure 1B]. Furthermore, we observed no time-dependent effect of the treatment on cell morphology and viability compared to untreated cells [Figure 1B]. These results suggest that IL-17A and Poly(I:C) did not affect cell death in placental cell lines. However, we observed the mRNA expression of various cytokines by IL-17A or Poly(I:C) treatment. IL-17 receptor mRNA and protein expression, IL-17RA and IL-17RC, were increased by IL-17A cytokine treatment [Figure 1D]. This result means that IL-17RA and IL-17RC genes are expressed in placental cell lines, and IL-17A binds to the receptor and affects various regulatory mechanisms. In addition, IL-17A treatment increased IFNγ mRNA expression, and Poly(I:C) treatment increased TNFα mRNA expression, but the mRNA expression of IL-6 was not affected by IL-17A or Poly(I:C) treatment compared with the control [Figure 1C,D]. These results suggest the possibility of affecting placental function through a signaling mechanism through IL-17A-IL-17R binding. In addition, we observed that PPARγ, a transcription factor related to glucose metabolism, was increased by IL-17A treatment, especially in the JEG-3 cells. These results suggest that IL-17A is associated with glucose metabolism in trophoblast cells.

**Figure 2:**
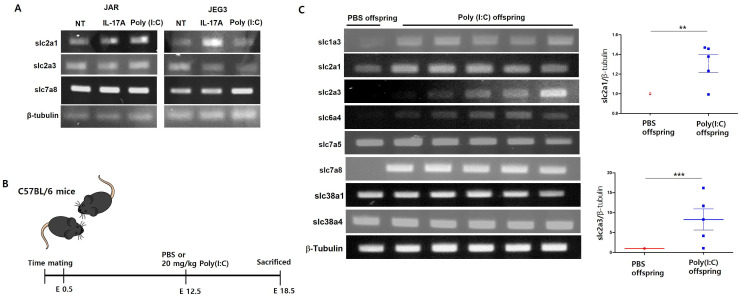
Glucose transporter mRNA expression in MIA-induced offspring’s placenta. SLC2A1, SLC2A3, and SLC7A8 mRNA expression in placenta cell treatment with 100 ng/mL IL-17A or 100 µg/mL (**A**). Scheme of maternal immune activation (MIA) mice models (**B**). SLC1A3, SLC2A1, SLC2A3, SLC6A4, SLC7A5, SLC38A1, and SLC38A4 mRNA expression from MIA-induced offspring’s placenta at E18.5 (**C**). ** *p* < 0.001, *** *p* < 0.001 as calculated by one-way ANOVA with Tukey’s test.

Also, according to SFARI Gene (https://www.sfari.org/resource/sfari-gene/, accessed on 2 June, 2023), it has been reported that the expression of several transporters is inhibited in the brains of individuals with autism spectrum disorder (ASD) [Table 3]. So, we investigated whether IL-17A treatment affects the expression of the glucose transporter in placental cells. It was observed that the mRNA expression of SLC2A1 (solute carrier family 2 member 1), known as glucose transporter 1 (Glut1), was increased by IL-17A treatment; however, SLC2A3 (solute carrier family 2 member 3), also known as glucose transporter 3 (Glut3), was decreased in JEG-3 trophoblast cells. However, it was observed that it had no effect on the mRNA expression of the amino acid transporter SLC7A8 compared with the control [Figure 2A].

**Figure 3:**
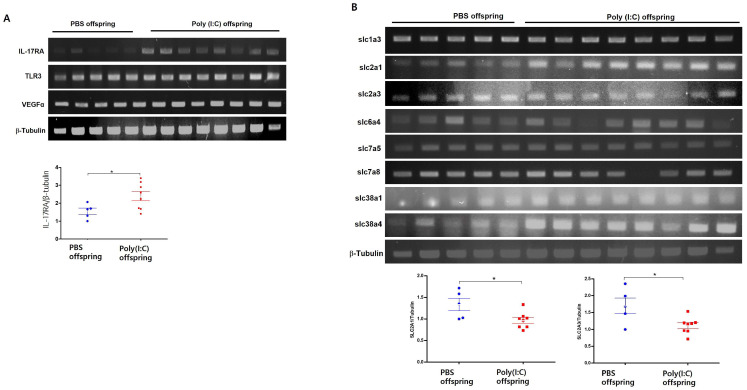
Glucose transporter mRNA expression in MIA-induced offspring’s brains. Activation of IL-17RA, TLR3, VEGFα, and ZO-1 mRNA expression in MIA offspring’s brains (**A**). SLC1A3, SLC2A1, SLC2A3, SLC6A4, SLC7A5, SLC38A1, and SLC38A4 mRNA expression from MIA-induced offspring’s brains at E18.5 (**B**). * *p* < 0.05, as calculated by one-way ANOVA with Tukey’s test.

Several reports have shown that increased maternal IL-17A affects fetal brain development in the Poly(I:C)-induced maternal immune activation (MIA) model at E12.5 in pregnant mice [Figure 2B]. So, we isolated the fetal brain of MIA-induced offspring and placenta tissue on embryonic day (E18.5) and examined the transporter’s expression. E18.5 in mice is a crucial stage in fetal brain development, corresponding roughly to the late third trimester in human gestation [15]. During this period, important stages of cerebral cortical development include neurogenesis, synaptogenesis, and glial cell formation. Therefore, we aimed to observe changes in the expression of glucose transporters in the placenta and fetal brain due to exposure to IL-17A or poly(I:C) at E12.5, when cerebral cortex development begins. IL-17RA mRNA expression was increased compared with PBS control in placentas isolated from MIA-induced E18.5 [Figure 2C]. We observed increased expression of glucose transporters SLC2A1 and SLC2A3 in MIA-induced placentas. This is consistent with the increase in transporter gene expression resulting from IL-17A treatment in the placental cell line JEG-3. It is well known that Glut1 expression is upregulated whenever the concentration of glucose is low. Interestingly, there was also no difference in the expression of glutamate transporter genes (SLC38A1 and SLC38A4) upon MIA induction. And several studies have reported increased IL-17RA expression in MIA-induced offspring’s brains. In this study, we also confirmed that IL-17RA mRNA expression was increased in MIA-induced offspring’s brains. Additionally, the mRNA expression of VEGFα, known as a gene that stimulates angiogenesis, and ZO-1, known as a tight junction protein, were also observed to be increased in the MIA-induced offspring’s brains [Figure 3A]. Then, we examined the gene expression of various transporters in the offspring’s brains. The expression of glucose transporters was observed to decrease mRNA expression in the brains of MIA-induced offspring. SLC2A1 and SLC2A3 mRNA expression were significantly reduced compared to the brains of the PBS control offspring. However, it did not affect the expression of some amino acid transporters, such as SLC7A8. Interestingly, mRNA expression of SLC38A1 and SLC38A4, known glutamate transporters, was significantly increased in the brains of MIA-induced offspring [Figure 3B]. This is thought to be related to a decrease in glucose transporters.

## 4. Discussion

Glucose serves as the primary source of energy for the fetus, obtained through the maternal circulatory system and placenta. Numerous GLUT isoforms have been identified in the placenta [6,7]. Glut1, prevalent in human tissue, undergoes regulation influenced by blood glucose concentration, cell signaling mechanisms, hormones, and other factors. [16]. This suggests that a substantial amount of glucose is required during the fetal brain developmental period. An increase in IL-17A, indicative of maternal immune activation (MIA), suggests that more energy is required for fetal brain development; however, a clear correlation has not yet been identified. Glut1 expression exhibits a two-fold increase in the late secondary trimester, remaining unaltered until term [17]. The placenta, a heterogeneous fetal organ encompassing various cell types, plays a critical role in supporting fetal development. Syncytiotrophoblasts and cytotrophoblasts, two key types within the placenta, are situated on the maternal surface. Trophoblasts, which include both syncytiotrophoblasts and cytotrophoblasts, are crucial for facilitating communication between the fetus and mother and contribute significantly to nutrient and gas exchanges [18]. JEG-3 cells, renowned for their capacity to produce placental hormones and mimic the biological functions of syncytiotrophoblasts, [19,20], exhibited an increase in Glut1 mRNA expression upon IL-7A treatment compared to the JAR placental cell line. Additionally, Glut1 mRNA expression was found to be heightened in the placenta of offspring induced by MIA. These findings suggest a potential regulatory role of IL-17A in Glut1 expression within placental cells. Regulation of trophoblast Glut1 protein expression involves several regulatory elements. Reports indicate that maternal conditions, such as diabetes accompanied by hyperglycemia or chronic hypoxia, can lead to diminished Glut1 expression, resulting in reduced glucose delivery to the fetus and potential fetal demise [21,22,23]. Furthermore, IL-17A deficiency has been associated with severe adult-onset obesity in vivo [24]. In vitro studies have shown that IL-17A inhibits insulin-induced glucose uptake in adipocytes [25] and increases the expression of various metabolic genes [26], highlighting its multifaceted role in metabolic regulation. This study investigated the impact of IL-17A on transporter expression in placenta cell lines and MIA animal models. While direct effects on cell growth in placental cell lines or observable morphological differences in MIA-induced placental tissues were not observed (results not presented), the modulation of trophoblast function was evident through altered transporter gene expression. Additionally, in vitro investigations suggested that IL-17A treatment of JEG-3 cells regulates their invasive capacity [27,28]. Nonetheless, future research is imperative to comprehensively understand the functional implications of IL-17A treatment on placental cells and placental tissues, and its subsequent effects on fetal health. Further exploration of IL-17A’s role in placental function and fetal development will contribute to our understanding of pregnancy-related pathologies and potential therapeutic interventions. Additionally, exploring potential therapeutic interventions to modulate IL-17A or glucose levels to improve pregnancy outcomes could be a promising research direction. This could potentially identify new ways to manage pregnancy complications associated with immune dysregulation and metabolic disorders. For this purpose, the aromatic recombinant CYP-19 Cre transgenic mouse model can be widely used for placental gene inactivation [29]. Placenta-specific inactivation of IL-17R and/or Glut1 may offer the possibility to study their effects on fetal brain development.

## Figures and Tables

**Table 1:** List of human primers used in this study.

	Forward	Reverse
IL-17a	TAATGGCCCTGAGGAATGGC	AGGAAGCCTGAGTCTAGGGG
IL-17RA	TGCCCTGTGGGTGTACTGGT	GGAGGCAGGCCATCGGTGTA
IL-17RC	CTGCCCTTGTGCAGTTTGG	CAGATTCGTACCTCACTCCCTA
IL-6	TACCCCCAGGAGAAGATTCC	AGTGCCTCTTTGCTGCTTTC
IFNγ	AACTGTCGCCAGCAGCTAAA	TTGGCCCCTGAGATAAAGCC
TNFα	GACAAGCCTGTAGCCCATGT	GGAGGTTGACCTTGGTCTGG
SLC2A1	GCTTATGGGCTTCTCCAAACT	GGTGACACCTCTCCCACATAC
SLC2A3	GATCGGCTCTTTCCAGTTTG	CAATCATGCCACCAACAGAG
SLC7A8	CCAGTGTGTTGGCCATGATC	TGCAACCGTTACCCCATAGAA
ACTIN	CAAGAGATGGCCACGGCTGCT	TCCTTCTGCATCCTGTCGGCA

**Table 2:** List of mouse primers used in this study.

	Forward	Reverse
IL-17RA	AGATGCCAGGATCCTGTACC	CACAGTCACAGCGTGTCTCA
TLR3	CCTCCAACTGTCTACCAGTTCC	GCCTGGCTAAGTTATTGTGG
VEGFα	GCCTTGTTCAGAGCGGAGAA	TGTCAACGGTGACGATGATG
ZO-1	AAAACGCTCTACAGGCTCCC	GGCCTGGGCTGGATCATAAC
SLC1A3	GAGGCAGCTCCCAGGTTTA	GATTACTCCCCCGCAGCCTA
SLC2A1	GTGACAAGACACCCGAGGAG	CTGTCGGTTCGGAAGAGGTC
SLC2A3	TCTCCTAAGTCACCGAGCCA	GCTACCTCAAACACACCCGA
SLC6A4	TGGTTTGTGCTCATCGTGGT	GCATTTCCTTCACGTCGCTG
SLC7A5	CTGGGTGAGACAGTTGGGTC	GCCATTCCAGTAGACACCCC
SLC7A8	GGGTGTCTACTGGCAACACA	CGGGTCCTTGACAGGAGTAG
SLC38A1	TCTGACTTCGGTGACACTGC	TGCATCCTCCTCTCCCATGA
SLC38A4	GCGGCCCTCTTTGGTTATCT	AGCGATCCCGAAATGCTTCA
TUBULIN	GAAGGACAGGAATGGGAACA	AGGTGTCTGGGAAGCTGAGA

**Table 3:** List of transporter genes associated with autism spectrum disorder (ASD).

Transporter	Function	Gene_Dpi
SLC1A3	Excitatory amino acid transporter, high-affinity glutamate transporter family	0.577
SLC6A4	Neurotransmitter serotonin from synaptic spaces	0.808
SLC6A8	Neurotransmitter transporter, creatines	0.846
SLC9A9	Sodium/proton exchanger, cation homeostasis	0.577
SLC25A12	Calcium-binding mitochondrial carrier protein	0.462
SLC40A1	Involved in iron export from duodenal epithelial cells	0.731

## Data Availability

The data used to support the findings of this study are included within the article.

## References

[1] Gude N.M., Roberts C.T., Kalionis B., King R.G. (2004). Growth and function of the normal human placenta. Thromb. Res..

[2] Burton G.J., Fowden A.L. (2015). The placenta: A multifaceted, transient organ. Philos. Trans. R. Soc. B Biol. Sci..

[3] Yockey L.J., Iwasaki A. (2018). Interferons and Proinflammatory Cytokines in Pregnancy and Fetal Development. Immunity.

[4] Robertson S.A., Seamark R.F., Guilbert L.J., Wegmann T.G. (1994). The Role of Cytokines in Gestation. Crit. Rev. Immunol..

[5] Zenobia C., Hajishengallis G. (2015). Basic biology and role of interleukin-17 in immunity and inflammation. Periodontology 2000.

[6] Wong H., Hoeffer C. (2018). Maternal IL-17A in autism. Exp. Neurol..

[7] Cornelius D.C., Hogg J.P., Scott J., Wallace K., Herse F., Moseley J., Wallukat G., Dechend R., LaMarca B. (2013). Administration of inter-leukin-17 soluble receptor C suppresses TH17 cells, oxidative stress, and hypertension in response to placental ischemia during pregnancy. Hypertension.

[8] Kuwabara T., Ishikawa F., Kondo M., Kakiuchi T. (2017). The Role of IL-17 and Related Cytokines in Inflammatory Autoimmune Diseases. Mediat. Inflamm..

[9] Ito M., Nakashima A., Hidaka T., Okabe M., Bac N.D., Ina S., Yoneda S., Shiozaki A., Sumi S., Tsuneyama K. (2010). A role for IL-17 in induction of an inflammation at the fetomaternal interface in preterm labour. J. Reprod. Immunol..

[10] Nelson D.M., Myatt L. (2020). The human placenta in health and disease. Obstet. Gynecol. Clin..

[11] Kalhan S.C., D’Angelo L.J., Savin S.M., Adam P.A. (1979). Glucose production in pregnant women at term gestation: Sources of glucose for human fetus. J. Clin. Investig..

[12] Castillo-Castrejon M., Powell T.L. (2017). Placental Nutrient Transport in Gestational Diabetic Pregnancies. Front. Endocrinol..

[13] Brett K.E., Ferraro Z.M., Yockell-Lelievre J., Gruslin A., Adamo K.B. (2014). Maternal–Fetal Nutrient Transport in Pregnancy Pathologies: The Role of the Placenta. Int. J. Mol. Sci..

[14] Al-Bishri W.M. (2023). Glucose transporter 1 deficiency, AMP-activated protein kinase activation and immune dysregulation in autism spectrum disorder: Novel biomarker sources for clinical diagnosis. Saudi J. Biol. Sci..

[15] Chen V.S., Morrison J.P., Southwell M.F., Foley J.F., Bolon B., Elmore S.A. (2017). Histology atlas of the developing prenatal and postnatal mouse central nervous system, with emphasis on prenatal days E7. 5 to E18. 5. Toxicol. Pathol..

[16] Pragallapati S., Manyam R. (2019). Glucose transporter 1 in health and disease. J. Oral Maxillofac. Pathol. JOMFP.

[17] Illsley N.P., Baumann M.U. (2020). Human placental glucose transport in fetoplacental growth and metabolism. Biochim. Biophys. Acta (BBA) Mol. Basis Dis..

[18] Tashev S.A., Parsons D., Hillman C., Harris S., Lofthouse E.M., Goggin P., Chatelet D.S., Cleal J.K., Smyth N., Palaiologou H. (2021). Folding of the syncytiotrophoblast basal plasma membrane increases the surface area available for exchange in human placenta. Placenta.

[19] Karteris Zachariades E., Foster H., Goumenou A., Thomas P., Rand-Weaver M., Karteris E. (2011). Expression of membrane and nuclear progesterone receptors in two human placental choriocarcinoma cell lines (JEG-3 and BeWo): Effects of syncytialization. Int. J. Mol. Med..

[20] Drwal E., Rak A., Gregoraszczuk E. (2018). Co-culture of JEG-3, BeWo and syncBeWo cell lines with adrenal H295R cell line: An al-ternative model for examining endocrine and metabolic properties of the fetoplacental unit. Cytotechnology.

[21] Gaither K., Quraishi A.N., Illsley N.P. (1999). Diabetes alters the expression and activity of the human placental GLUT1 glucose trans-porter. J. Clin. Endocrinol. Metab..

[22] Takagi H., King G.L., Aiello L.P. (1998). Hypoxia upregulates glucose transport activity through an adenosine-mediated increase of GLUT1 expression in retinal capillary endothelial cells. Diabetes.

[23] Das U.G., Sadiq H.F., Soares M.J., Hay W.W., Devaskar S.U. (1998). Time-dependent physiological regulation of rodent and ovine pla-cental glucose transporter (GLUT-1) protein. Am. J. Physiol.-Regul. Integr. Comp. Physiol..

[24] Kim H.Y., Lee H.J., Chang Y.J., Pichavant M., Shore S.A., Fitzgerald K.A., Iwakura Y., Israel E., Bolger K., Faul J. (2014). In-terleukin-17–producing innate lymphoid cells and the NLRP3 inflammasome facilitate obesity-associated airway hyperreac-tivity. Nat. Med..

[25] Hou S., Jiao Y., Yuan Q., Zhai J., Tian T., Sun K., Chen Z., Wu Z., Zhang J. (2018). S100A4 protects mice from high-fat diet-induced obesity and inflammation. Mod. Pathol..

[26] Qu Y., Zhang Q., Ma S., Liu S., Chen Z., Mo Z., You Z. (2016). Interleukin-17A Differentially Induces Inflammatory and Metabolic Gene Expression in the Adipose Tissues of Lean and Obese Mice. Int. J. Mol. Sci..

[27] Zhu X.W., Mulcahy L.A., Mohammed R.A.A., Lee A.H.S., Franks H.A., Kilpatrick L., Yilmazer A., Paish E.C., Ellis I.O., Patel P.M. (2008). IL-17 expression by breast-cancer-associated macrophages: IL-17 promotes invasiveness of breast cancer cell lines. Breast Cancer Res..

[28] Fu B., Tian Z., Wei H. (2014). TH17 cells in human recurrent pregnancy loss and pre-eclampsia. Cell. Mol. Immunol..

[29] Wenzel P.L., Leone G. (2007). Expression of Cre recombinase in early diploid trophoblast cells of the mouse placenta. Genes.

